# Effects of repetitive transcranial magnetic stimulation on upper-limb and finger function in stroke patients: A systematic review and meta-analysis of randomized controlled trials

**DOI:** 10.3389/fneur.2022.940467

**Published:** 2022-07-29

**Authors:** Gengbin Chen, Tuo Lin, Manfeng Wu, Guiyuan Cai, Qian Ding, Jiayue Xu, Wanqi Li, Cheng Wu, Hongying Chen, Yue Lan

**Affiliations:** ^1^Department of Rehabilitation Medicine, Guangzhou First People's Hospital, School of Medicine, South China University of Technology, Guangzhou, China; ^2^Postgraduate Research Institute, Guangzhou Sport University, Guangzhou, China; ^3^Department of Rehabilitation Medicine, the Second Affiliated Hospital, School of Medicine, South China University of Technology, Guangzhou, China; ^4^Guangzhou Key Laboratory of Aging Frailty and Neurorehabilitation, Guangzhou, China

**Keywords:** repetitive transcranial magnetic stimulation, stroke, meta-analysis, hand, upper limb, review

## Abstract

**Background:**

Repetitive transcranial magnetic stimulation (rTMS) is a promising intervention for stroke rehabilitation. Several studies have demonstrated the effectiveness of rTMS in restoring motor function. This meta-analysis aimed to summarize the current evidence of the effect of rTMS in improving upper limb function and fine motor recovery in stroke patients.

**Methods:**

Three online databases (Web of Science, PubMed, and Embase) were searched for relevant randomized controlled trials. A total of 45 studies (combined *n* = 2064) were included. Random effects model was used for meta-analysis and effect size was reported as standardized mean difference (SMD).

**Results:**

rTMS was effective in improving fine motor function in stroke patients (SMD, 0.38; 95% CI 0.19–0.58; *P* = 0). On subgroup analyses, for post-stroke functional improvement of the upper extremity, bilateral hemisphere stimulation was more effective than unilateral stimulation during the acute phase of stroke, and a regimen of 20 rTMS sessions produced greater improvement than <20 sessions. In the subacute phase of stroke, affected hemispheric stimulation with a 40-session rTMS regimen was superior to unaffected hemispheric stimulation or bilateral hemispheric stimulation with <40 sessions. Unaffected site stimulation with a 10-session rTMS regimen produced significant improvement in the chronic phase compared to affected side stimulation and bilateral stimulation with >10 rTMS sessions. For the rTMS stimulation method, both TBS and rTMS were found to be significantly more effective in the acute phase of stroke, but TBS was more effective than rTMS. However, rTMS was found to be more effective than TBS stimulation in patients in the subacute and chronic phases of stroke. rTMS significantly improved upper limb and fine function in the short term (0–1-month post-intervention) and medium term (2–5 months), but not for upper limb function in the long term (6 months+). The results should be interpreted with caution due to significant heterogeneity.

**Conclusions:**

This updated meta-analysis provides robust evidence of the efficacy of rTMS treatment in improving upper extremity and fine function during various phases of stroke.

**Systematic Review Registration:**

https://inplasy.com/inplasy-2022-5-0121/, identifier: INPLASY202250121.

## Introduction

In Europe, more than 1 million new cases of stroke are reported each year. Owing to the progressive population aging, the absolute number of stroke victims is projected to increase in the near future (1). Approximately, 50%−80% of stroke survivors experience upper limb dysfunction (2). Recovery of upper limb function is associated with improvement in activities of daily living and mental health (3). However, very few stroke survivors show complete recovery of upper limb function 6 months after stroke (4). In addition, rehabilitation has a limited impact on the recovery of hand motor function.

Repetitive transcranial magnetic stimulation (rTMS) is a non-invasive, non-painful therapeutic technique for cortical excitability regulation. Cortical excitability can be increased by high-frequency transcranial magnetic stimulation (rTMS) or intermittent Theta-burst stimulation (TBS), whereas it can be suppressed by low-frequency rTMS or continuous TBS (5). A large number of studies have investigated the efficacy of rTMS for post-stroke rehabilitation. Moreover, several literature reviews and meta-analyses have attempted to synthesize the available evidence of the efficacy of rTMS for post-stroke upper extremity dysfunction.

An early meta-analysis of small randomized controlled trials (RCTs) suggested that TMS improves motor outcomes in paralyzed upper limbs and hands after stroke (6, 7). However, there is considerable variability among the RCTs with respect to the outcomes of TMS, which may be attributable to the methodological differences (8, 9). Moreover, previous reviews (10) have not assessed the effect of variables such as baseline injury level, stimulation of the hemisphere, etc. However, it is known that these factors have an effect on the effectiveness of TMS (11, 12). Moreover, previous meta-analyses have not systematically explored the effect of various recovery factors in different phases of stroke.

The current work aimed to identify the factors that influence the efficacy of rTMS in improving upper limb function in different phases of stroke (stimulation location, baseline impairment level, number of treatment sessions, stimulation method, rTMS frequency), while incorporating new high-quality evidence to update an earlier review on the same topic (6). In addition, we aimed to identify the short-, medium-, and long-term effects of rTMS intervention on post-stroke upper limb and hand function. Only high-quality sham-controlled randomized trials were included in the current analysis to maximize the value and interpretability of the results.

## Methods

### Protocol and search strategy

The Preferred Reporting Items for Systematic Reviews and Meta-Analyses (PRISMA) standards were followed for conducting this meta-analysis (13).

To find relevant research published in English, a thorough search of the literature was undertaken utilizing three online databases (Web of Science, PubMed, and Embase). The search terms were revised for each database and are presented in Supplementary Table S1. The latest research was completed on February 12th, 2022. Additionally, to find additional relevant research, the references in the included papers and the reference lists of prior systematic reviews were manually examined.

### Eligibility criteria

The following were among the study's inclusion criteria: (1) randomized controlled trials of rTMS in adult patients (age ≥18 years) diagnosed with stroke based on relevant clinical examinations; (2) intervention group received rTMS alone or rTMS in combination with an additional intervention, while the control group received sham rTMS (SrTMS) or no rTMS; (3) minimum sample size: 5 patients; (4) primary outcome measure: the Fugl-Meyer Assessment Upper Extremity (FMA-UE); secondary outcomes: hand functional dexterity assessed using box and block test, nine-hole peg test, and Purdue pegboard test; (5) methodological quality rated as high according to the PEDro (Physiotherapy Evidence Database) scale (see below, quality analysis). RCTs with a crossover design were excluded.

### Data extraction

Two researchers (CGB, DQ) independently extracted the data and evaluated the quality of the eligible studies. A third independent investigator (LY) resolved any inconsistencies. Information relating to the name of the first author, year of publication, count of participants, patient characteristics (stroke stage and baseline impairment level), treatment parameters (type of rTMS and intensity, number of pulses and sessions, stimulated site), outcome measurements, and the duration of follow-up. Mean difference (MD) and Standard deviation (SD) between the pre- and post-intervention outcome indicators (rTMS and SrTMS) for each group were taken from each study. If no numerical data were provided, these data were extracted from the figures using GetData Graph Digitizer 2.25 based on the Cochrane Handbook for Systematic Reviews of Interventions (14).

### Data synthesis and analysis

To investigate the impact of rTMS on upper limb and hand function in different phases of stroke (acute [<1 month] vs. subacute [1 month to 6 months] vs. chronic [>6 months]) (15), we followed the recommendations for upper extremity and hand function assessment in stroke rehabilitation according to the latest stroke guidelines (16). The results of box and block test (BBT), nine-hole peg test (NHPT), and purdue pegboard test (PPT) were used to assess fine motor and manual skills, while the results of upper extremity Fugl-Meyer Assessment (FMA-UE) were pooled to evaluate upper limb motor function.

To investigate the effect of rTMS in patients with different levels of baseline injury, three subgroups were established based on the initial impairment level (17) (Supplementary Table S2): (1) mild (FMA-UE score: 43–66), (2) moderate (FMA-UE score: 29–42), and (3) severe (FMA-UE score: 0–28) baseline impairment. The weighted mean of the baseline data for the rTMS and SrTMS groups was used to compute the mean baseline FMA-UE score for each research. To identify other potential influences on motor recovery, subgroup analyses were also done depending on the stimulated sites (affected vs. unaffected sides vs. bilateral), number of treatment sessions, stimulation method (rTMS vs. TBS), and rTMS frequency (1 Hz vs. 3–10 Hz vs. 20 Hz). Finally, we explored the effectiveness of rTMS intervention on upper extremity and manual dexterity after a period of time. Since the current review did not clearly delineate the follow-up time, the effects were divided into short-term (0–1 months after intervention), medium-term (2–5 months), and long-term (6+ months).

All analyses were performed using the StataMP14.0 software. To compare the results, the effect size and accompanying 95 percent confidence intervals (CIs) were employed. The I^2^ statistic and the Cochrane's Q test were applied to evaluate heterogeneity among the included studies. The random-effects model was applied to enable the generalization of the results beyond the included studies. *P*-values < 0.05 were considered indicative of statistical significance. The effect size was expressed as the standardized mean difference (SMD).

### Quality assessment

The quality of the included studies was independently assessed by two assessors using the PEDro scale (18, 19). The scale consists of 11 items, and each of the 10 scored quality criteria was scored as 1 (criterion fulfilled) or 0 (criterion not fulfilled). The individual item scores were added to obtain the total score for each study. Since the first item is not included in the total result, the maximal total score for each study is 10/10, which indicates high methodological quality. Studies with a total score ≤ 6 were excluded.

## Results

### Characteristics of the RCTs

**Figure 1:**
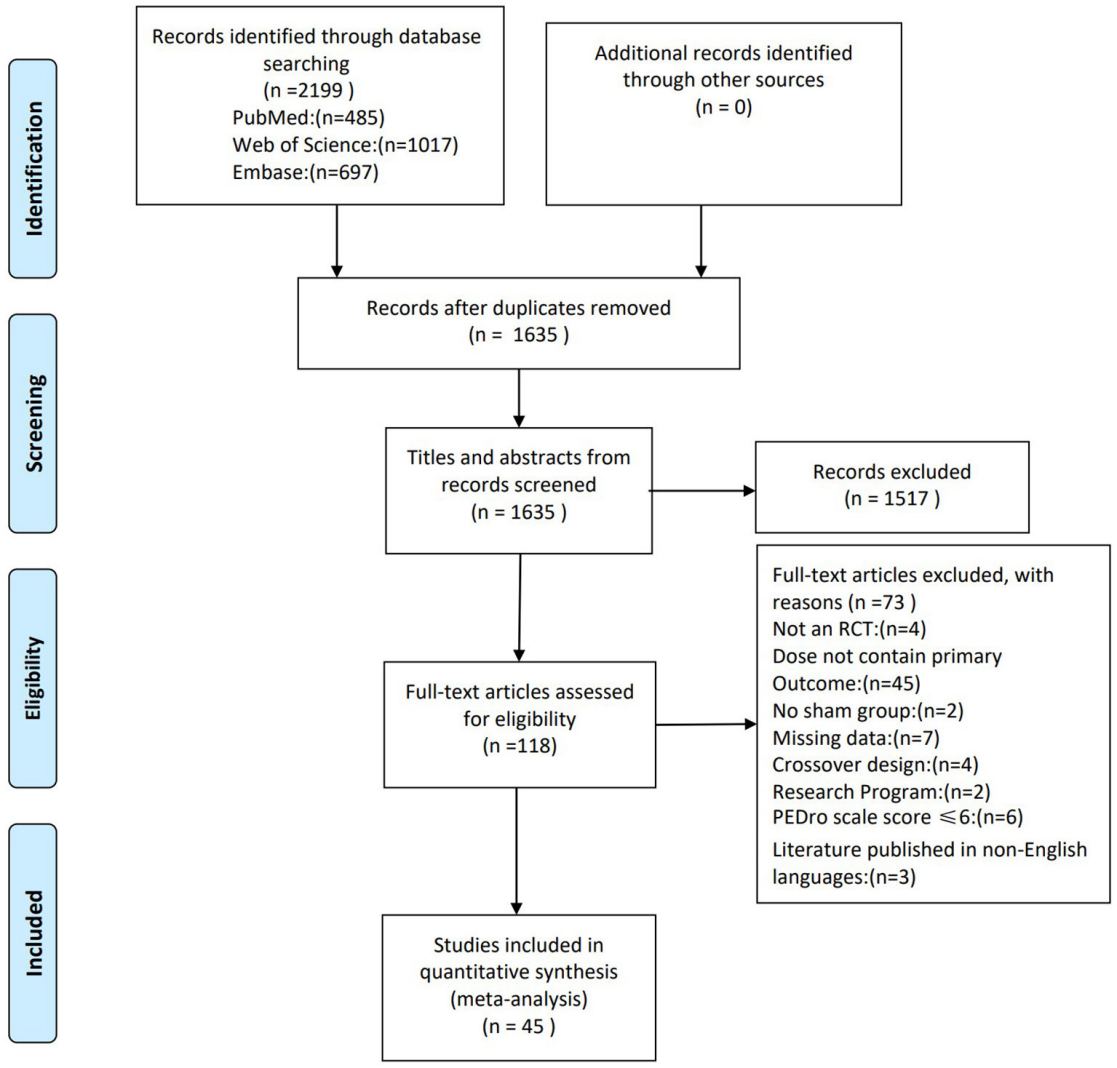
PRISMA flow chart on selection and inclusion of studies.

A total of 2,199 articles were retrieved on database search (Figure 1). Of these, 564 duplicates were excluded Screening of the remaining 1,635 papers was done based on the study titles and abstracts, which led to the exclusion of 1,517 papers. Full text of the remaining 118 papers were assessed for eligibility and the risk of bias was assessed using PEDro scale. Of these 73 studies did not qualify the eligibility criteria: non-RCT (4 studies); inconsistent outcome measures (45 studies); lack of control group (2 studies); missing data (7 studies); crossover design (4 studies), research program (2 studies); PEDro score ≤ 6 (6 studies), and non-English language publication (3 studies).

Finally, this meta-analysis included 45 high-quality randomized controlled trials with a total population of 2064 stroke patients. Supplementary Table S2 shows the characteristics of the included studies. Sub-studies were identified in sixteen studies. These studies included 2 (20–31), 3 (32–34) and 4 experimental groups (35) (each group differed in terms of patient characteristics or TMS protocol) and indicated the FMA-UE scores respectively, for each subgroup separately. The mean age of patients varied from 51.0 to 74.0 years, and the mean age of controls ranged from 50.5 to 75.0 years. On average, the studies scored 8.42 ± 0.92 (mean ± SD) on the PEDro scale, which indicates high methodological quality (Table 1).

**Table 1:** Assessment of risk of bias in the included studies.

**References**	**Criteria**											**Total**
	**1**	**2**	**3**	**4**	**5**	**6**	**7**	**8**	**9**	**10**	**11**	
Askin et al. (36)	Y	1	0	1	0	0	1	1	1	1	1	7
Barros et al. (59)	Y	1	1	1	1	1	1	1	1	1	1	10
Bonin et al. (60)	Y	1	1	1	1	1	1	1	1	1	1	10
Cha et al. (37)	Y	1	1	1	1	0	1	1	1	1	1	9
Chang et al. (38)	Y	1	0	1	1	0	1	1	1	1	1	8
Chen et al. (32)	Y	1	1	1	1	0	1	1	1	1	1	9
Chen et al. (39)	Y	1	0	1	1	0	1	1	1	1	1	8
Chen et al. (41)	Y	1	0	1	1	0	1	1	1	1	1	8
Chervyakov et al. (33)	Y	1	1	1	1	0	1	0	1	1	1	8
Di Lazzaro et al. (63)	Y	1	0	1	1	0	1	1	1	1	1	8
Di Lazzaro et al. (40)	Y	1	0	1	1	0	1	1	1	1	1	8
Du et al. (20)	Y	1	1	1	1	0	1	0	1	1	1	8
Du et al. (21)	Y	1	0	1	0	0	1	1	1	1	1	7
Du et al. (22)	Y	1	1	1	1	1	1	1	1	1	1	10
Fregni et al. (42)	Y	1	0	1	1	0	1	1	1	1	1	8
Guan et al. (51)	Y	1	1	1	1	1	1	1	1	1	1	10
Harvey et al. (61)	Y	1	0	1	1	1	1	1	1	1	1	9
Hosomi et al. (53)	Y	1	0	1	1	1	1	1	1	1	1	9
Hsu et al. (52)	Y	1	0	1	1	0	1	1	1	1	1	8
Jil et al. (43)	N	1	0	1	1	0	1	1	1	1	1	8
Juan et al. (23)	Y	1	1	1	1	0	1	1	1	1	1	9
Khan et al. (50)	Y	1	1	1	0	0	1	1	1	1	1	8
Khedr et al. (24)	Y	1	0	1	1	0	1	1	1	1	1	8
Kim et al. (25)	Y	1	0	1	1	0	1	1	1	1	1	8
Kuzu et al. (26)	Y	1	0	1	1	0	1	1	1	1	1	8
Li et al. (27)	Y	1	0	1	1	0	0	1	1	1	1	7
Long et al. (28)	Y	1	0	1	1	0	1	1	1	1	1	8
Luk et al. (44)	Y	1	1	1	1	1	1	1	1	1	1	10
Malcolm et al. (45)	Y	1	0	1	1	0	1	1	1	1	1	8
Matsuura et al. (46)	Y	1	0	1	1	0	1	1	1	1	1	8
Meng et al. (29)	Y	1	0	1	1	0	1	1	1	1	1	8
Haghighi et al. (47)	Y	1	0	1	1	0	1	1	1	1	1	8
Qin et al. (56)	Y	1	0	1	1	0	0	1	1	1	1	7
Rose et al. (62)	Y	1	0	1	1	1	1	1	1	1	1	9
Sharma et al. (58)	Y	1	1	1	1	1	1	1	1	1	1	10
Sung et al. (34)	Y	1	1	1	1	0	1	1	1	1	1	9
Wang et al. (35)	Y	1	1	1	1	0	1	1	1	1	1	9
Yang et al. (54)	Y	1	0	1	1	0	0	1	1	1	1	7
Seniów et al. (57)	Y	1	1	1	1	1	0	1	1	1	1	9
Gottlieb et al. (55)	Y	1	1	1	1	1	1	1	1	1	1	10
Higgins et al. (48)	Y	1	0	1	1	0	1	1	1	1	1	8
kim et al. (30)	Y	1	1	0	0	0	1	1	1	1	1	7
Watanabe et al. (31)	Y	1	0	1	1	0	1	1	1	1	1	8
Özkeskin et al. (64)	Y	1	1	1	1	0	1	1	1	1	1	9
Kim et al. (49)	Y	1	1	1	1	0	1	1	1	1	1	9

*Criteria numbers: 1, eligibility criteria; 2, random allocation; 3, concealed allocation; 4, similar groups at baseline; 5, blinding subjects; 6, blinding therapists; 7, blinding assessors; 8, outcome obtained in more than 85% of the subjects; 9, intention-to-treat analysis; 10, between-group statistical comparisons; 11, point estimates and measures of variability*.

### Effects of rTMS or sham stimulation on hand function recovery

The effect of rTMS on manual dexterity was assessed by pooling post-intervention data from 17 studies (24, 30, 36–49); hand function was measured by box and block test (36–39, 41, 43–45, 47–49), nine-hole peg test (30, 39, 40, 44), and purdue pegboard test (24, 42, 46). The results of pooled data showed a significant improvement in the treatment group (SMD, 0.38; 95% CI, 0.19–0.58, *P* = 0) (Supplementary Figure 1A and Table 2).

**Table 2:** Subgroup analysis: treatment vs. control.

	**Studies (N)**	**SMD (95% CI)**	***P***	**I**^2^ **(%)**	***P*** **(heterogeneity)**
**Hand function**					
Overall	17	0.38 (0.19, 0.58)	0	0	0.839
Stage of stroke					
Acute	6	0.27 (−0.02, 0.56)	0.068	0	0.581
Subacute	3	0.69 (0.22, 1.16)	0.004	0	0.571
Chronic	8	0.38 (0.07, 0.69)	0.018	0	0.845
Follow–up time					
Short–term					
(0–1 month after intervention) Medium–term	6	0.35 (0.04, 0.66)	0.026	0	0.986
(2–5 months after intervention)	6	0.40 (0.05, 0.75)	0.023	0	0.975
**Upper extremity**					
**Acute phase**					
Stimulation site					
Affected side	7	0.71 (0.24, 1.19)	0.003	66.8	0.006
Unaffected side	8	0.51 (0.02, 1.01)	0.043	75.7	0
Bilateral	2	5.99 (1.88, 10.09)	0.004	93.9	0
Baseline impairment					
Mild baseline impairment	1	0.61 (−0.29, 1.51)	0.183		
Moderate baseline impairment	6	0.44 (−0.01, 0.89)	0.053	52.7	0.061
Severe baseline impairment	10	1.59 (0.68, 2.49)	0.001	93.4	0
Treatment sessions					
5–sessions	6	0.35 (0.09, 0.62)	0.009	0	0.461
10–sessions	5	0.50 (−0.00, 1.01)	0.052	55.8	0.060
12–15 sessions	3	1.39 (−0.98, 3.75)	0.250	94.7	0
20–sessions	3	3.73 (1.22, 6.24)	0.004	96	0
Stimulation					
TBS rTMS rTMS frequencyx Moderate (3–10 Hz) Low (1Hz)	2 15 6 8	3.08 (1.25, 4.91) 0.88 (0.35, 1.41) 0.61 (0.15, 1.07) 0.51 (0.02, 1.01)	0.001 0.001 0.01 0.043	75.5 88.2 65 75.7	0.043 0 0.014 0
**Subacute phase**					
Stimulation site					
Affected side	7	0.52 (0.01, 1.02)	0.044	74.4	0.001
Unaffected side	9	0.45 (0.13, 0.77)	0.005	56.6	0.018
Bilateral	3	0.42 (−0.04, 0.87)	0.072	4.3	0.352
Baseline impairment					
Mild baseline impairment	3	0.76 (−0.15, 1.67)	0.103	79.9	0.007
Moderate baseline impairment	5	0.22 (−0.20, 0.64)	0.301	56.6	0.056
Severe baseline impairment	11	0.54 (0.23, 0.86)	0.001	55.2	0.013
Treatment sessions					
5–sessions	1	−0.12 (−0.75, 0.51)	0.711		
10–sessions	14	0.56 (0.27, 0.85)	0	62.8	0.001
15–sessions	1	−0.04 (−0.66, 0.58)	0.894		
20–sessions	2	0.25 (−0.24, 0.75)	0.313	0	0.832
40–sessions	1	0.88 (0.23, 1.52)	0.008		
Stimulation					
TBS rTMS rTMS frequency High (20 Hz) Moderate (3–10Hz) Low (1Hz)	1 15 2 4 9	−0.18 (−0.89, 0.53) 0.52 (0.24, 0.79) 0.77 (0.05, 1.49) 0.59 (−0.16, 1.34) 0.45 (0.13, 0.77)	0.619 0 0.037 0.122 0.005	63.6 43 82.1 56.6	0 0.185 0.001 0.018
**Chronic phase**					
Stimulation site					
Affected side	6	0.07 (−0.26, 0.41)	0.67	0	0.99
Unaffected side	14	0.42 (0.14, 0.69)	0.003	60.7	0.002
Bilateral	4	0.56 (−0.14, 1.25)	0.116	69.4	0.02
Baseline impairment					
Moderate baseline impairment	11	0.34 (0.03, 0.65)	0.03	51.7	0.023
Severe baseline impairment	13	0.39 (0.11, 0.67)	0.006	48.6	0.025
Treatment sessions					
10–sessions	13	0.32 (0.08, 0.55)	0.008	0	0.680
15–sessions	3	0.73 (−0.15, 1.62)	0.102	78.1	0.01
16–sessions	1	0.15 (−0.75, 1.05)	0.743		
18–sessions	2	−0.03 (−0.31, 0.25)	0.846	0	0.707
20–sessions	3	0.43 (−0.03, 0.89)	0.065	70.9	0.008
Stimulation					
TBS rTMS rTMS frequency Moderate (3–10Hz) Low (1Hz)	6 16 1 13	0.05 (−0.29, 0.40) 0.48 (0.21, 0.76) 0.23 (−0.59, 1.06) 0.43 (0.14, 0.72)	0.76 0.001 0.581 0.004	0 64.5 63.5	0.994 0 0.001
**Overall**					
**Follow–up time**					
Short–term					
(0–1 month after intervention) Medium–term	14	0.27 (0.04, 0.51)	0.023	34.5	0.099
(2–5 months after intervention) Long–term	23	1.23 (0.74, 1.73)	0	90.4	0
(6+ months after intervention)	3	1.61 (−0.43, 3.65)	0.121	95.9	0

*SMD, standardized mean difference; CI, confidence interval*.

Subgroup analysis showed significant effect sizes for recovery of hand function in subacute phase (37, 44, 47): SMD = 0.69, 95% CI 0.22–1.16, *P* = 0.004, chronic phase (36, 39–43, 45, 48): SMD = 0.38, 95% CI 0.07–0.69, *P* = 0.018, follow-up duration [short-term (24, 30, 40, 42, 49): SMD = 0.35, 95% CI 0.04–0.66, *P* = 0.026; medium-term (24, 38, 40, 44, 45): SMD = 0.40, 95% CI 0.05–0.75, *P* = 0.023] (Supplementary Figure 1C and Table 2), but not the acute phase (24, 30, 38, 46, 49): (SMD = 0.27, 95% CI −0.02–0.56, *P* = 0.068) (Supplementary Figure 1B and Table 2).

### Effect of rTMS or sham stimulation on FMA-UE in patients with acute phase stroke

**Figure 2:**
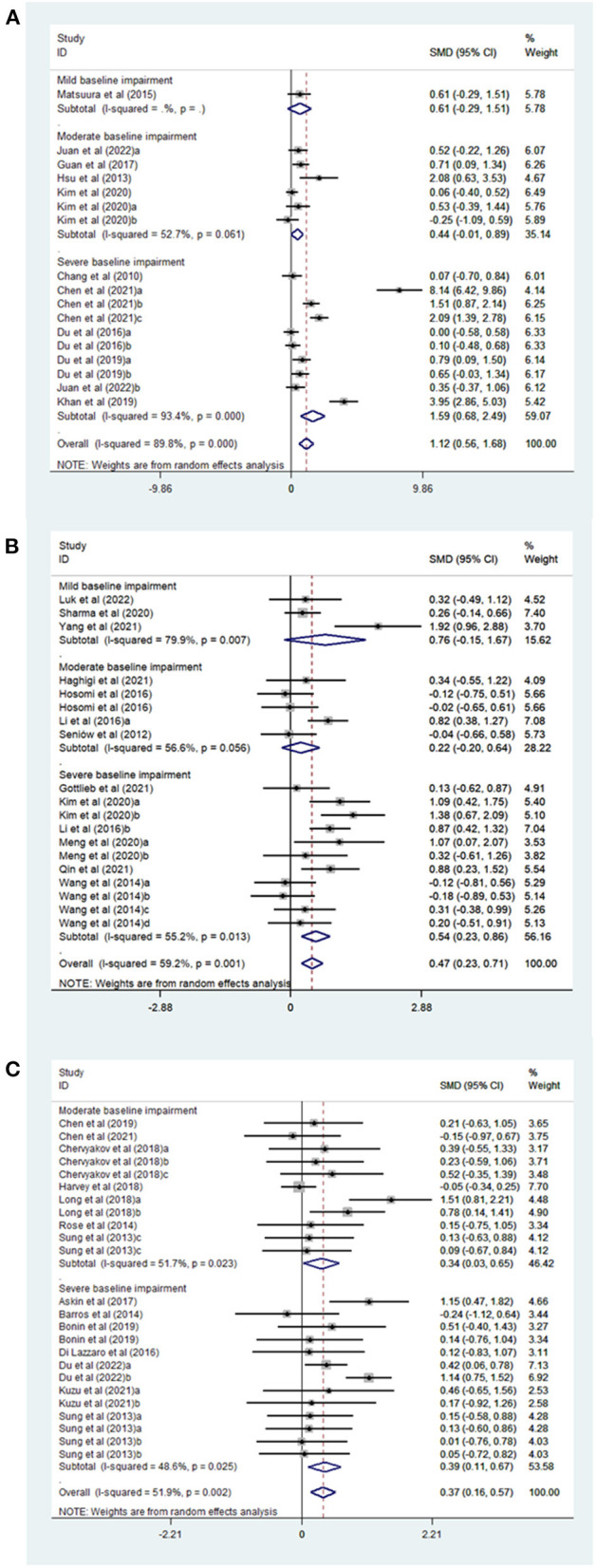
**(A)** Forest plot of FMA-UE in patients with acute phase stroke disaggregated by baseline impairment level compared with controls. **(B)** Forest plot of FMA-UE in patients with Subacute phase stroke disaggregated by baseline impairment level compared with controls. **(C)** Forest plot of FMA-UE in patients with chronic phase stroke disaggregated by baseline impairment level compared with controls.

The following subgroup analyses were done to investigate the effect of relevant factors on clinical outcomes, as shown in Table 2. Subgroup analysis based on the stimulation sites indicated that upper-extremity outcomes of patients with acute stroke were significantly improved by bilateral (32, 50) (SMD, 5.99; 95% CI 1.88–10.09; *P* = 0.004), affected side (20, 22, 23, 32, 38, 51, 52) (SMD, 0.71; 95% CI 0.24–1.19; *P* = 0.003) and unaffected side stimulation (20, 22, 23, 25, 32, 46, 49) (SMD, 0.51, 95% CI 0.02–1.01; *P* = 0.043, Supplementary Figure 2A). On subgroup analysis based on baseline impairment level, a significant effect was noted in favor of the rTMS group in patients with severe baseline impairment (20, 22, 23, 32, 38, 50) (SMD, 1.59; 95% CI 0.68–2.49; *P* = 0.001) but not in those with mild baseline impairment (46) (SMD, 0.61; 95% CI −0.29–1.51; *P* = 0.183) or moderate baseline impairment (23, 30, 49, 51, 52) (SMD, 0.44; 95% CI −0.01–0.89; *P* = 0.053, Figure 2A). In a subgroup analysis based on the rTMS method, a significant effect of TBS (50, 52) (SMD, 3.08; 95% CI 1.25–4.91; *P* = 0.001) and rTMS (20, 22, 23, 30, 32, 38, 46, 49, 51) (SMD, 0.88; 95% CI 0.35–1.41; *P* = 0.001) was noted (Supplementary Figure 2B). Additionally, subgroup analysis based on rTMS frequency showed that 1 Hz (20, 22, 23, 30, 32, 46, 49) (SMD, 0.51; 95% CI 0.02–1.01; *P* = 0.043) and 3–10 Hz (20, 22, 23, 32, 38, 51) (SMD, 0.61; 95% CI 0.15–1.07; *P* = 0.01) produced a significant effect (Supplementary Figure 2C).

Seventeen studies were divided into five subgroups based on the number of treatment sessions: 5 sessions, 10 sessions, 12–15 sessions, and 20 sessions. The effect sizes were as follows: (SMD, 0.35; 95% CI 0.09–0.62; *P* = 0.009) for 5 sessions (20, 22, 23); (SMD, 0.50; 95% CI −0.00–1.01; *P* = 0.052) for 10 sessions (38, 46, 49, 51, 52); (SMD, 1.39; 95% CI −0.98–3.75; *P* = 0.25) for 12–15 sessions (30, 50) and (SMD, 3.73; 95% CI 1.22–6.24; *P* = 0.004, Supplementary Figure 2D) for 20 sessions (32).

### Effect of rTMS or sham stimulation on FMA-UE in patients with subacute phase stroke

Subgroup analyses revealed significant improvement on FMA-UE in patients with post-stroke upper limb dysfunction in relation to stimulation site [affected site (25, 27, 35, 47, 53, 54) SMD, 0.52, 95% CI 0.01–1.02; *P* = 0.044; unaffected side (25, 27, 29, 35, 44, 55–58): SMD, 0.45, 95% CI 0.13–0.77; *P* = 0.005], but not the bilateral stimulation (29, 35) (SMD, 0.42, 95% CI −0.04–0.87; *P* = 0.072) (Supplementary Figure 3A and Table 2). A significant effect was observed in favor of the rTMS group in patients with severe baseline impairment (25, 27, 29, 35, 55, 56) (SMD, 0.54; 95% CI 0.23–0.86; *P* = 0.001), but not in patients with mild baseline impairment (44, 54, 58) (SMD, 0.76; 95% CI −0.15–1.67; *P* = 0.103) or moderate baseline impairment (27, 47, 53, 57) (SMD, 0.22; 95% CI −0.20–0.64; *P* = 0.301) (Figure 2B and Table 2). In a subgroup analysis based on the rTMS method, a significant effect was noted in the rTMS group (25, 27, 29, 35, 44, 47, 53–58) (SMD, 0.52; 95% CI 0.24–0.79; *P* = 0) but not in TBS group (35) (SMD, −0.18; 95% CI −0.89–0.53; *P* = 0.619) (Supplementary Figure 3B and Table 2). Additionally, subgroup analysis based on rTMS frequency showed that 20 Hz (25, 47) (SMD, 0.77; 95% CI 0.05–1.49; *P* = 0.037) and 1 Hz rTMS (25, 27, 29, 35, 44, 55–58) (SMD, 0.45; 95% CI 0.13–0.77; *P* = 0.005) were able to produce greater effects than 3–10 Hz rTMS (27, 53, 54) (SMD, 0.59; 95% CI −0.16–1.34; *P* = 0.122) (Supplementary Figure 3C and Table 2).

Nineteen studies were divided into five subgroups based on the number of treatment sessions: 5 sessions, 10 sessions, 15 sessions, 20 sessions, and 40 sessions. The effect sizes were as follows: (SMD, −0.12; 95% CI −0.75–0.51; *P* = 0.711) for 5 sessions (53); (SMD, 0.56; 95% CI 0.27–0.85; *P* = 0) for 10 sessions (25, 27, 29, 35, 44, 47, 53–55, 58); (SMD, −0.04; 95% CI −0.66–0.58; *P* = 0.894) for 15 sessions (57); (SMD, 0.25; 95% CI −0.24–0.75; *P* = 0.313) for 20 sessions (35) and (SMD, 0.88; 95% CI 0.23–1.52; *P* = 0.008) for 40 sessions (56) (Supplementary Figure 3D and Table 2).

### Effect of rTMS or sham stimulation on FMA-UE in patients with chronic phase stroke

Subgroup analyses revealed significant improvement on FMA-UE in individuals with post-stroke upper limb dysfunction in relation to stimulation site [unaffected side (21, 26, 28, 33, 34, 36, 59–62): SMD, 0.42, 95% CI 0.14–0.69; *P* = 0.003], but not the affected site (33, 34, 39, 41, 63) (SMD, 0.07, 95% CI−0.26–0.41; *P* = 0.67) or bilateral stimulation (28, 33, 34) (SMD, 0.56, 95% CI -−0.14–1.25; *P* = 0.116) (Supplementary Figure 4A and Table 2). Transcranial magnetic stimulation had a significant effect in patients with both severe baseline impairment (21, 26, 34, 36, 59, 60, 63) (SMD, 0.39; 95% CI 0.11–0.67; *P* = 0.006) and moderate baseline impairment (SMD, 0.34; 95% CI 0.03–0.65; *P* = 0.03) (Figure 2C and Table 2). In a subgroup analysis based on the rTMS method, a significant effect was noted in the rTMS group (21, 26, 28, 33, 34, 36, 59–62) (SMD, 0.48; 95% CI 0.21–0.76; *P* = 0.001) but not in TBS group (26, 34, 39, 41, 63) (SMD, 0.05; 95% CI −0.29–0.40; *P* = 0.76) (Supplementary Figure 4B and Table 2). Further, subgroup analysis based on rTMS frequency showed that 1 Hz rTMS (21, 26, 28, 33, 34, 36, 59–62) (SMD, 0.43; 95% CI 0.14–0.72; *P* = 0.004) produced better results than 3–10 Hz rTMS (33) (SMD, 0.23; 95% CI −0.59–1.06; *P* = 0.581) (Supplementary Figure 4C and Table 2).

Twenty-four studies were sorted into five subgroups depending on the number of treatment sessions: 10 sessions, 15 sessions, 16 sessions, 18 sessions, and 20 sessions. The effect sizes were as follows: (SMD, 0.32; 95% CI 0.08–0.55; *P* = 0.008) for 10 sessions (26, 33, 34, 36, 41, 59, 60, 63); (SMD, 0.73; 95% CI −0.15–1.62; *P* = 0.102) for 15 sessions (28, 39); (SMD, 0.15; 95% CI −0.75–1.05; *P* = 0.743) for 16 sessions (62); (SMD, −0.03; 95% CI −0.31–0.25; *P* = 0.846) for 18 sessions (60, 61) and (SMD, 0.43; 95% CI−0.03–0.89; *P* = 0.065) for 20 sessions (21, 34) (Supplementary Figure 4D and Table 2).

### Short-term, medium-term and long-term effects of transcranial magnetic stimulation or sham stimulation on FMA-UE

The post-intervention outcome is represented here by the timing of the assessment rounds: short-term, intermediate, and long-term effects. Fourteen studies (20, 26, 30, 49, 51–53, 59, 61, 63, 64) had short-term follow-up within 0–1 month after the end of the intervention, 23 studies (20, 22, 23, 28, 31, 32, 35, 38, 44, 50, 51, 57, 61, 63, 64) had intermediate follow-up assessments lasting 2–5 months after the end of intervention, and 3 studies (50, 51, 61) had long-term assessments. A significant benefit was found at short-term follow-up (SMD, 0.27; 95% CI 0.04–0.51; *P* = 0.023) and medium-term follow-up (SMD, 1.23; 95% CI, 0.74–1.73; *P* = 0), whereas no significant effect was observed at long-term follow-up (SMD, 1.61; 95% CI −0.43–3.65; *P* = 0.121) (Supplementary Figure 5 and Table 2).

### Sensitivity analysis

Sensitivity analyses were performed on selected studies to identify the potential influence of outliers on the overall results. The results showed no significant influence of any individual study on the results of meta-analysis (Supplementary Figure 6).

## Discussion

rTMS and iTBS are used to treat post-stroke upper extremity and hand dysfunction; however, a meta-analysis of recent RCTs is lacking. Therefore, the aim of this study was to evaluate the effect of rTMS on the upper limb and hand function at different stroke stages in order to explore the appropriate stimulation modality. Based on the available evidence, our meta-analysis further validated the significant therapeutic effect of this procedure. On subgroup analysis, for upper limb recovery in the acute phase of stroke, rTMS protocol with bilateral hemispheric stimulation, 20 treatment sessions, was more effective than unilateral hemispheric stimulation, <20 treatment sessions. To recover the upper limb function in the subacute phase of stroke, affected hemispheric stimulation, 40 sessions of rTMS protocol, was better than unaffected hemispheric stimulation or bilateral hemispheric stimulation, <40 sessions. To rehabilitate the upper limbs in the chronic phase of the stroke, unaffected hemispheric stimulation, 10 sessions of rTMS protocol was found to be better than the affected hemispheric stimulation or bilateral hemispheric stimulation for >10 sessions. Regarding the effect of rTMS on post-stroke hand dysfunction, significant effects were achieved in both the subacute and chronic phases of stroke.

As mentioned previously, high-frequency rTMS and iTBS can promote the stimulated area, and low-frequency rTMS and cTBS can inhibit the stimulated area (5). In our meta-analysis, subgroup analysis showed the superiority of bilateral rTMS over the affected side rTMS and unaffected side rTMS in the acute phase of stroke. Affected rTMS was more effective than unaffected side rTMS and bilateral rTMS in the subacute phase of stroke. Unaffected side rTMS was found superior to affected side rTMS and bilateral rTMS in the chronic phase. Only 9 trials included in our meta-analysis had used bilateral rTMS, and the most common choices for bilateral rTMS included affected hemisphere M1 and unaffected hemisphere M1. The most common choices for bilateral rTMS included affected hemisphere M1 and unaffected hemisphere M1, whereas affected hemisphere M1 and unaffected hemisphere M1 were applied in 20 and 31 trials, respectively. The preferential selection of primary motor cortex may be attributable to the critical role of this area in locomotion.

Our subgroup analysis of the number of sessions showed that rTMS for 20 sessions was more effective than stimulation for <20 sessions in the acute phase of stroke, and that rTMS for 40 sessions was more effective than stimulation for <40 sessions in the subacute phase. However, rTMS for 10 sessions was more effective than stimulation for >10 sessions in the chronic phase. Our results are not very consistent with those of the previous meta-analysis (65), in which stimulation for 5 sessions was found to produce a better effect than 1, 10, and 15–16 sessions of rTMS. We included additional RCTs conducted in recent years. Studies included in our meta-analysis showed multiple sessions of rTMS applied during the acute and subacute phases of stroke. Thus, our results suggest a longer-term treatment effect to a certain extent.

As for the severity of upper extremity impairment in patients, previous studies (7) have reported more significant hand function recovery after LF-rTMS for mild-moderate stroke. In our subgroup analysis, patients with severe baseline injury benefited more from rTMS intervention for post-stroke upper extremity function in the acute or subacute period than those with mild or moderate baseline impairment. However, rTMS for upper extremity dysfunction in the chronic phase of stroke was found to produce equally significant outcomes in individuals with moderate and severe injury. Our results are not very consistent with the existing knowledge. The meta-analysis by Van et al. (66) showed that transcranial magnetic direct current stimulation significantly improved patients with mild and moderate impairment, but not those with severe impairment. We then further analyzed the existing studies and found that in the acute phase of stroke, the protocol of bilateral and affected rTMS stimulation was mainly applied for patients with severe baseline injury, whereas patients with moderate baseline injury were administered unaffected rTMS. In the subacute phase, studies of patients with severe baseline injury mainly entailed application of the unaffected and bilateral rTMS protocol, while the affected rTMS protocol was mainly applied for patients with moderate baseline injury. The difference in results may be due to different treatment modalities, and the fact that only four trials included patients with mild baseline injury. Therefore, further studies are required before concluding which baseline level of impairment is suitable for rTMS intervention in different stroke phases. However, there is no doubt that rTMS was found to significantly improve the severity of baseline injury in patients with different stroke phases.

As for the rTMS stimulation method, previous studies (65) have reported equally significant effects of rTMS and TBS on upper extremity functional recovery in stroke patients. In our subgroup analysis, in the acute phase of stroke, significant effects were observed with both TBS and rTMS, but TBS was better than rTMS. However, rTMS was found to be more effective than TBS in patients in the subacute and chronic phases of stroke.

With respect to rTMS stimulation frequency, in former studies (65, 67), high-frequency rTMS (>1 Hz) and low-frequency rTMS (≤ 1 Hz) were found to produce equally significant effects, and high-frequency rTMS was found to induce greater functional recovery of the upper limb than sham stimulation. We further investigated the appropriate frequency for patients in different phases of stroke; on subgroup analysis, 1 Hz and 3–10 Hz rTMS showed equally significant effects in the acute phase of stroke, while 1 Hz and 20 Hz rTMS had more significant effects than 3–10 Hz rTMS in the subacute phase of stroke, and 1 Hz rTMS had better effects than 3–10 Hz rTMS in the chronic phase.

Previous studies (68) have suggested that patients with acute and subacute stroke may not benefit from non-invasive brain stimulation as much as patients with chronic stroke. As more new high methodological quality RCTs are published, we conducted a meta-analysis to investigate the efficacy of rTMS in improving post-stroke hand dysfunction and observed significant effects in subacute and chronic patients, but the effect size was greater in subacute patients.

On subgroup analysis, we found significant effects of rTMS intervention in the short term (0–1 month after intervention) and medium term (2–5 months). Our result was largely consistent with that of the previous meta-analysis (65), which demonstrated long-term beneficial effect of rTMS on upper extremity motor function recovery, as assessed ≥ 1 month after the last rTMS session. Furthermore, we included a number of new studies with a more careful delineation of the duration of post-intervention follow-up and found no significant effects after 6 months of intervention with rTMS in the upper limb after stroke.

The current meta-analysis includes the most recently published randomized controlled trials based on strict inclusion criteria. Thus, our study adds value to the published literature and provides high-level evidence in the field. Furthermore, building on the existing evidence, we have conducted the most comprehensive investigation of influencing factors by performing eighteen subgroup analyses disaggregated by stroke phase and different baseline injury level, which may help identify the optimal stimulation pattern and appropriate treatment indications, and thus promote this non-invasive therapy in the long term.

Certain limitations of our study should be recognized. First, although we performed subgroup analysis based on different stroke phases, some subgroups comprised only one or two studies. Therefore, to obtain more robust evidence, further RCTs are needed. Second, to explore the optimal rTMS parameters for patients with different stroke stages, we extracted the mean time after stroke onset for each trial as a characteristic for categorizing the trials into stroke stages, and some trials may involve patients with different stroke stages. Third, the protocols included in the study showed significant differences. rTMS pulse counts ranged from 200 to 2000, and although the subgroup analyses improved homogeneity and comparability within subgroups, some subgroups remained quite heterogeneous. Therefore, due caution should be exercised while interpreting our results. Finally, some of our subgroup analyses had relatively small sample sizes, and therefore further clinical trials are required to validate the robustness of our results.

## Conclusion

This meta-analysis further demonstrates the role of rTMS therapy for post-stroke upper limb and hand dysfunction. In the acute phase of stroke, bilateral hemispheric stimulation with a 20-treatment rTMS regimen was more effective than unilateral hemispheric stimulation with <20 treatments. In the subacute phase of stroke, affected hemispheric stimulation with a 40-treatment rTMS regimen was superior to unaffected hemispheric stimulation or bilateral hemispheric stimulation with <40 treatments. In the chronic phase of stroke, unaffected hemispheric stimulation with a 10-session rTMS regimen was superior to affected hemispheric stimulation or bilateral hemispheric stimulation with >10 sessions. In addition, rTMS significantly improved upper limb function in patients with severe baseline impairment across stroke phases. Both TBS and rTMS were found to be significantly effective in the acute phase of stroke, but TBS was more effective than rTMS. However, rTMS was found to be more effective than TBS stimulation in the subacute and chronic phases of stroke. Subgroup analysis of rTMS on post-stroke hand dysfunction showed significant effects in both the subacute and chronic phases of stroke. rTMS intervention had significant effects on upper limb and hand motor function in stroke patients in the short term (0–1-month post-intervention) and in the medium term (2–5 months). However, given the relatively small sample sizes in some subgroups, the results must be interpreted with caution.

## Data availability statement

Publicly available datasets were analyzed in this study. This data can be found here: Pubmed, Web of Science, and Embase.

## Author contributions

GCh and TL conceived the review and wrote the manuscript. GCh, TL, QD, MW, and GCa researched the literature. YL revised the manuscript for intellectual content. All authors contributed to manuscript revision and read and approved the submitted version.

## Funding

This research was supported by grant 81772438, 81974357, 82072548, and 82102678 from the National Science Foundation of China and grant 202206010197 from Guangzhou Municipal Science and Technology Program.

## Conflict of interest

The authors declare that the research was conducted in the absence of any commercial or financial relationships that could be construed as a potential conflict of interest.

## Publisher's note

All claims expressed in this article are solely those of the authors and do not necessarily represent those of their affiliated organizations, or those of the publisher, the editors and the reviewers. Any product that may be evaluated in this article, or claim that may be made by its manufacturer, is not guaranteed or endorsed by the publisher.
