# Supplementary material for: Effects of repetitive transcranial magnetic stimulation on upper-limb and finger function in stroke patients: A systematic review and meta-analysis of randomized controlled trials
Source: Front Neurol. 2022 Jul 29;13:940467. doi: 10.3389/fneur.2022.940467 (PMC9372362; doi:10.3389/fneur.2022.940467)
Supplement: Supplementary file 1 [file Data_Sheet_1.pdf]

Supplementary Table 1 Search engine and query

Search engine	Search query	Date of search
Pubmed	((((stroke[MeSH Terms]) OR (stroke) OR (cerebrovascular accident)) AND ((transcranial magnetic stimulation[MeSH Terms]) OR (transcranial magnetic stimulation) OR (TMS) OR (repetitive transcranial magnetic stimulation) OR (rTMS) OR (theta burst stimulation) OR (TBS))) AND ((Manual dexterity) OR (motor movement) OR (hand dexterity) OR (motor skills) OR (Upper limb) OR (upper extremity[MeSH Terms]) OR (upper extremity) OR (hand[MeSH Terms]) OR (hand) OR (hands))) AND ((randomized controlled trial[Publication Type]) OR (controlled clinical trial[Publication Type]) OR (randomized[Title/Abstract]) OR (placebo[Title/Abstract]) OR (randomly[Title/Abstract]) OR (trial[Title/Abstract]) OR (groups[Title/Abstract]))	12-Feb-22
Embase	(stroke OR (cerebrovascular AND accident)) AND (tms OR (transcranial AND magnetic AND stimulation) OR (repetitive AND transcranial AND magnetic AND stimulation) OR rtms OR (theta AND burst AND stimulation) OR tbs) AND (hand OR (manual AND dexterity) OR (motor AND movement) OR (hand AND dexterity) OR (motor AND skills) OR (upper AND limb) OR (upper AND extremity) OR hands) AND ('randomized controlled trial':it OR 'controlled clinical trial':it OR randomized:ab,ti OR placebo:ab,ti OR randomly:ab,ti OR trial:ab,ti OR groups:ab,ti)	12-Feb-22
Web of science	(((TS=(stroke OR cerebrovascular accident)) AND TS=(transcranial magnetic stimulation OR TMS OR repetitive transcranial magnetic stimulation OR rTMS OR theta burst stimulation OR TBS)) AND TS=(Manual dexterity OR motor movement OR hand dexterity OR motor skills OR Upper limb OR upper extremity OR hand OR hands)) AND TS=(randomized controlled trial OR controlled clinical trial OR randomized OR placebo OR randomly OR trial OR groups)	12-Feb-22

Supplementary Table 2 Characteristics of the included studies

References	No. of participants (Exp/Ctr)	Mean age (years) (Exp/Ctr)	Mean time Post-stroke Stage	Baseline FMA-UE score Subgroup	rTMS protocol	Sessions	Stimulated site	Outcome measurement	Measure time
Askin et al(2017) [36]	40(20/20)	56.75/58.8	III	22.75 Severe	1Hz ,90%RMT,1200pulses	10 sessions	Unaffected side	FMA-UE BBT	Post-intervention
Barros et al(2014) [60]	20(10/10)	57.4/64.6	III	23.55 Severe	1Hz ,90%MT,1500pulses	10 sessions	Unaffected side	FMA-UE	Post-intervention, Follow-up,4w
Bonin et al(2019) [61]	19(9/10)	57.22/50.5	III	26.3 Severe	1Hz,100%RMT,1200pulses	18 sessions	Unaffected side	FMA-UE	After 10 sessions intervention Post-intervention
Cha et al (2016) [37]	25(15/10)	64.07/63.33	II		1Hz,90%MT,1200pulses	20 sessions	Right hemisphere, P3 10/20 EEG system	BBT	Post-intervention
Chang et al (2010) [38]	28(18/10)	56.4/57.0	I	20.19 Severe	10Hz,90%RMT,1000pulses	10 sessions	Affected side	FMA-UE BBT	Post-intervention, Follow-up,2m
Chen et al(2021)a [32]	50(25/25)	58/65	I	17.74 Severe	10Hz,90%RMT,600pulses +1Hz, 90%RMT, 600pulses	20 sessions	Bilateral	FMA-UE	Post-intervention, Follow-up,3 m
Chen et al(2021)b [32]	50(25/25)	62/65	I	19.86 Severe	10Hz,90%RMT,600pulses	20 sessions	Affected side	FMA-UE	Post-intervention, Follow-up,3 m
Chen et al(2021)c [32]	50(25/25)	63/65	I	18.71 Severe	1Hz,90%RMT,600pulses	20 sessions	Unaffected side	FMA-UE	Post-intervention, Follow-up,3 m
Chen et al(2021) [39]	23(12/11)	53.36/48.95	III	39.26 Moderate	iTBS,80%AMT,1200pulses	15 sessions	Affected side	FMA-UE BBT NHPT	Post-intervention
Chen et al(2019) [41]	22(11/11)	52.9/52.6	III	31.68 Moderate	iTBS, 80%AMT,600pulses	10 sessions	Affected side	FMA-UE BBT	Post-intervention
Chervyakov et al(2018)a [33]	18(8/10)	60.7/61.4	III	31.1 Moderate	10Hz,80%RMT,200pulses +1Hz ,100%RMT,1200pulses	10 sessions	Bilateral	FMA-UE	Post-intervention
Chervyakov et al(2018)b [33]	23(13/10)	58.6/61.4	III	32.87 Moderate	10Hz,80%RMT,200pulses	10 sessions	Affected side	FMA-UE	Post-intervention
Chervyakov et al(2018)c [33]	21(11/10)	54.2/61.4	III	31.22 Moderate	1Hz,100%RMT,1200pulses	10 sessions	Unaffected side	FMA-UE	Post-intervention
Di Lazzaro et al (2016) [64]	17(8/9)	57.88/56.78	III	13.47 Severe	cTBS,80%AMT,600pulses	10 sessions	Affected side	FMA-UE	Post-intervention, Follow-up,1m,3m
Di Lazzaro et al (2013) [40]	12(6/6)	59.5/57.5	III		cTBS,80%AMT,600pulses	10 sessions	Affected side	NHPT	Post-intervention, Follow-up 1m,3m
Du et al(2016)a [20]	46(23/23)	56.78/53.61	I	24.22 Severe	3Hz,80%-90%RMT,1200pulses	5 sessions	Affected side	FMA-UE	Post-intervention, Follow-up,1m,2m, 3m
Du et al(2016)b [20]	46(23/23)	56.78/53.61	I	24.28 Severe	1Hz,110%-120%RMT,1200pulses	5 sessions	Unaffected side	FMA-UE	Post-intervention, Follow-up,1m,2m, 3m
Du et al(2022)a [21]	120(60/60)	58.22/58.77	III	23.81 Severe	1Hz,90%RMT,20min	20 sessions	Unaffected side	FMA-UE	Post-intervention
Du et al(2022)b [21]	120(60/60)	59.05/57.2	III	23.85 Severe	1Hz,90%RMT,20min	20 sessions	Unaffected side	FMA-UE	Post-intervention

Supplementary Table 2 Characteristics of the included studies

References	No. of participants (Exp/Ctr)	Mean age (years) (Exp/Ctr)	Mean time Post-stroke Stage	Baseline FMA-UE score Subgroup	rTMS protocol	Sessions	Stimulated site	Outcome measurement	Measure time
Du et al(2019)a [22]	40(20/20)	54/56	I	27.5 Severe	10Hz,100%RMT,1200pulses	5 sessions	Affected side	FMA-UE	Post-intervention, Follow-up 3m
Du et al(2019)b [22]	40(20/20)	56/56	I	28 Severe	1Hz,100%RMT,1200pulses	5 sessions	Unaffected side	FMA-UE	Post-intervention, Follow-up 3m
Fregni et al(2006) [42]	15(10/5)	57.7/52.6	III		1Hz,100%MT,1200pulses	5 sessions	Unaffected side	PPT	Post-intervention, Follow-up,15d
Guan et al(2017) [51]	42(21/21)	59.7/57.4	I	39.15 Moderate	5Hz,120%MT,1000pulses	10 sessions	Unaffected side	FMA-UE	Post-intervention Follow-up, 15d,3m,6m,1Y
Harvey et al(2018) [62]	199(132/67)	59.2/57.6	III	34.34 Moderate	1Hz ,15min	18 sessions	Unaffected side	FMA-UE	Post-intervention, Follow-up,1m,3m,6m
Hosomi et al(2016) [54]	39(18/21)	62.4/63.2	II	36.50 Moderate	5Hz,90%RMT,500pulses	10 sessions	Affected side	FMA-UE	After 5 sessions intervention, Post-intervention, Follow-up 17d
Hsu et al(2013) [52]	12(6/6)	56.8/62.3	I	36.4 Moderate	iTBS,80%AMT,1200pulses	10 sessions	Affected side	FMA-UE	Post-intervention, Follow-up,1m
Jil et al(2014) [43]	23(12/11)	54.73/50.53	III		10Hz,15min	30 sessions	Affected side	BBT	Post-intervention
Juan et al(2022)a [23]	29(15/14)	51/52	I	29.14 Moderate	10Hz,100%RMT,1200pulses	5 sessions	Affected side	FMA-UE	Post-intervention, Follow-up,3m
Juan et al(2022)b [23]	31(17/14)	56/52	I	27.19 Severe	1Hz,100%RMT,1200pulses	5 sessions	Unaffected side	FMA-UE	Post-intervention, Follow-up,3m
Khan et al(2019) [50]	40(20/20)	63.55/64.60	I	14.6 Severe	iTBS,60%RMT,600pulses +cTBS,60%RMT,600pulses	12 sessions	Bilateral	FMA-UE	Post-intervention, Follow-up, 2m,5m,11m
Khedr et al(2009)a [24]	24(12/12)	59.0/60	I		3Hz,130%RMT,900pulses	5 sessions	Affected side	PPT	Post-intervention, Follow-up, 1m,2m,3m
Khedr et al(2009)b [24]	24(12/12)	54.7/60	I		1Hz,100%RMT,900pulses	5 sessions	Unaffected side	PPT	Post-intervention, Follow-up, 1m,2m,3m
Kim et al(2020)a [25]	40(20/20)	62.7/64.0	II	10.5 Severe	20Hz,90%RMT,1500pulses	10 sessions	Affected side	FMA-UE	Post-intervention
Kim et al(2020)b [25]	38(18/20)	61.8/64.0	II	10.2 Severe	1Hz,90%RMT,1500pulses	10 sessions	Unaffected side	FMA-UE	Post-intervention
Kuzu et al(2021)a [26]	13(7/6)	56.3/65	III	16.78 Severe	1Hz,90%RMT,1200pulses	10 sessions	Unaffected side	FMA-UE	Post-intervention, Follow-up, 1m
Kuzu et al(2021)b [26]	13(7/6)	61.3/65	III	19.31 Severe	cTBS,80%AMT,600pulses	10 sessions	Unaffected side	FMA-UE	Post-intervention, Follow-up, 1m
Li et al(2016)a [27]	85(43/42)	54.00/53.13	II	29.00 Moderate	10Hz,80%MT,1350pulses	10 sessions	Affected side	FMA-UE	Post-intervention

Supplementary Table 2 Continued

References	No. of participants (Exp/Ctr)	Mean age (years) (Exp/Ctr)	Mean time Post-stroke Stage	Baseline FMA-UE score Subgroup	rTMS protocol	Sessions	Stimulated site	Outcome measurement	Measure time
Li et al(2016)b [27]	84(42/42)	57.87/53.13	II	28.58 Severe	1Hz,80%MT,1000pulses	10 sessions	Unaffected side	FMA-UE	Post-intervention
Long et al (2018)a [28]	41(21/20)	55.9/56.85	III	34.92 Moderate	1Hz,90%RMT,1000pulses +10Hz,90%RMT,1000pulses	15 sessions	Bilateral	FMA-UE	Post-intervention, Follow-up, 3m
Long et al (2018)b [28]	41(21/20)	57/56.85	III	35.07 Moderate	1Hz,90%RMT,1000pulses	15 sessions	Unaffected side	FMA-UE	Post-intervention, Follow-up, 3m
Luk et al(2022) [44]	24(12/12)	67.3/65.1	II	47.75 Mild	1Hz,90%RMT,1200pulses	10 sessions	Unaffected side	FMA-UE BBT NHPT	Post-intervention, Follow-up, 10w
Malcolm(2007) [45]	19(9/10)	68.4/65.7	III		20Hz,90%MT,2000pulses	10 sessions	Affected side	BBT	Post-intervention, Follow-up 6m
Matsuura et al(2015) [46]	20(10/10)	72.2/74.7	I	50.4 Mild	1Hz,100%MT,1200pulses	5 sessions	Unaffected side	FMA-UE PPT	Post-intervention
Meng et al(2020)a [29]	18(10/8)	55.3/53.87	II	20.17 Severe	1Hz,100%RMT,1200pulses +iTBS,60%-80%RMT,1200pulses	10 sessions	Bilateral	FMA-UE	Post-intervention
Meng et al(2020)b [29]	18(10/8)	52.5/53.87	II	21.63 Severe	1Hz,100%RMT,1200pulses	10 sessions	Unaffected side	FMA-UE	Post-intervention
Haghighi et al(2021) [47]	20(10/10)	50.50/53.9	II	33.6 Moderate	20Hz,90%RMT,2000pulses	10 sessions	Affected side	FMA-UE BBT	Post-intervention
Qin et al(2021) [57]	41(23/18)	58.52/62.27	II	27.37 Severe	1Hz,90%MT,1200pulses	40 sessions	Unaffected side	FMA-UE	Post-intervention
Rose et al(2014) [63]	19(9/10)	64.7/64.6	III	39.18 Moderate	1Hz,100%RMT,1200pulses	16 sessions	Unaffected side	FMA-UE	Post-intervention
Sharma et al(2020) [59]	96(47/49)	54.85/52.89	II	45.25 Mild	1Hz,110%RMT,750pulses	10 sessions	Unaffected side	FMA-UE	Post-intervention
Sung et al(2013)a [34]	29(15/14)	62.3/63.1	III	27.66 Severe	1Hz,90%RMT,600pulses +iTBS,80%AMT,600pulses	20 sessions	Bilateral	FMA-UE	After 10 sessions intervention Post-intervention
Sung et al(2013)b [34]	26(12/14)	64.2/63.1	III	27.81 Severe	iTBS,80%AMT,600pulses	20 sessions	Affected side	FMA-UE	After 10 sessions intervention Post-intervention
Sung et al(2013)c [34]	27(13/14)	63.3/63.1	III	29.95 Moderate	1Hz,90%RMT,600pulses	20 sessions	Unaffected side	FMA-UE	After 10 sessions intervention Post-intervention
Wang et al(2014)a [35]	33(17/16)	62.2/62.5	II	26.2 Severe	1Hz,90%RMT,600pulses	10 sessions	Unaffected side	FMA-UE	Post-intervention
Wang et al(2014)b [35]	31(15/16)	63.1/62.5	II	27.26 Severe	iTBS,80%MT,600pulses	10 sessions	Affected side	FMA-UE	Post-intervention

Supplementary Table 2 Continued

References	No. of participants (Exp/Ctr)	Mean age (years) (Exp/Ctr)	Mean time Post-stroke Stage	Baseline FMA-UE score Subgroup	rTMS protocol	Sessions	Stimulated site	Outcome measurement	Measure time
Wang et al(2014)c [35]	33(17/16)	62.2/62.5	II	26.2 Severe	1Hz,90%RMT,600pulses, 10session; iTBS,80%MT, 600pulses ,10sessions	20 sessions	Bilateral	FMA-UE	Post-intervention Follow-up, 3m
Wang et al(2014)d [35]	31(15/16)	63.1/62.5	II	27.26 Severe	iTBS,80%MT,600pulses, 10 sessions; 1hz,90%RMT, 600pulses ,10 sessions	20 sessions	Bilateral	FMA-UE	Post-intervention Follow-up, 3m
Yang et al(2021) [55]	25(12/13)	64/64	II	47 Mild	5Hz,100%RMT,750pulses	10 sessions	Affected side	FMA-UE	Post-intervention
Senіów et al(2012) [58]	40(20/20)	63.5/63.4	II	38.2 Moderate	1Hz,90%RMT,1800pulses	15 sessions	Unaffected side	FMA-UE	Post-intervention Follow-up, 3m
Gottlieb et al(2021) [56]	28(14/14)	63.93/62.43	II	25.68 Severe	1Hz,100%RMT,1200pulses	10 sessions	Unaffected side	FMA-UE	Post-intervention
Higgins et al(2013) [48]	9(4/5)	74/60	III		1Hz,110%MT,1200pulses	8 sessions	Unaffected side	BBT	Post-intervention
Kim et al(2020)a [30]	20(8/12)	67.00/62.17	I	39.90 Moderate	1Hz,80%MT,1200pulses	15 sessions	Unaffected side	FMA-UE NHPT	Post-intervention Follow-up, 1m
Kim et al(2020)b [30]	22(11/11)	67.55/64.45	I	34.41 Moderate	1Hz,80%MT,1200pulses	15 sessions	Unaffected side	FMA-UE NHPT	Post-intervention Follow-up, 1m
Watanabe et al (2018)a [31]	13(7/6)	67.6/75.2	I	11.80 Severe	1Hz,110%RMT,1200pulses	10 sessions	Unaffected side	FMA-UE	Follow-up, 10w
Watanabe et al (2018)b [31]	14(8/6)	72.5/75.2	I	14.36 Severe	iTBS,80%RMT,600pulses	10 sessions	Affected side	FMA-UE	Follow-up, 10w
Özkeskin et al(2016) [65]	21(10/11)	55.7/65.54	III	39.52 Moderate	1Hz,90%RMT,1500pulses	10 sessions	Unaffected side	FMA-UE	Follow-up, 10d,1m,3m
Kim et al(2020) [49]	73(36/37)	61.2/62.9	I	40.69 Moderate	1Hz,100%RMT,1800pulses	10 sessions	Unaffected site	FMA-UE BBT	Post-intervention Follow-up, 1m

I , acute stroke; II ,subacute stroke; III ,chronic stroke; RMT, resting motor threshold; AMT, active motor threshold; Hz, hertz; m, months; w,weeks; d, days; y, years; Exp, experimental group; Ctr, control group; FMA-UE, Fugl-Meyer Assessment Upper Extremity Scale; BBT, Box and Block Test; NHPT, Nine Hole Peg Test; PPT, Purdue Pegboard Test.

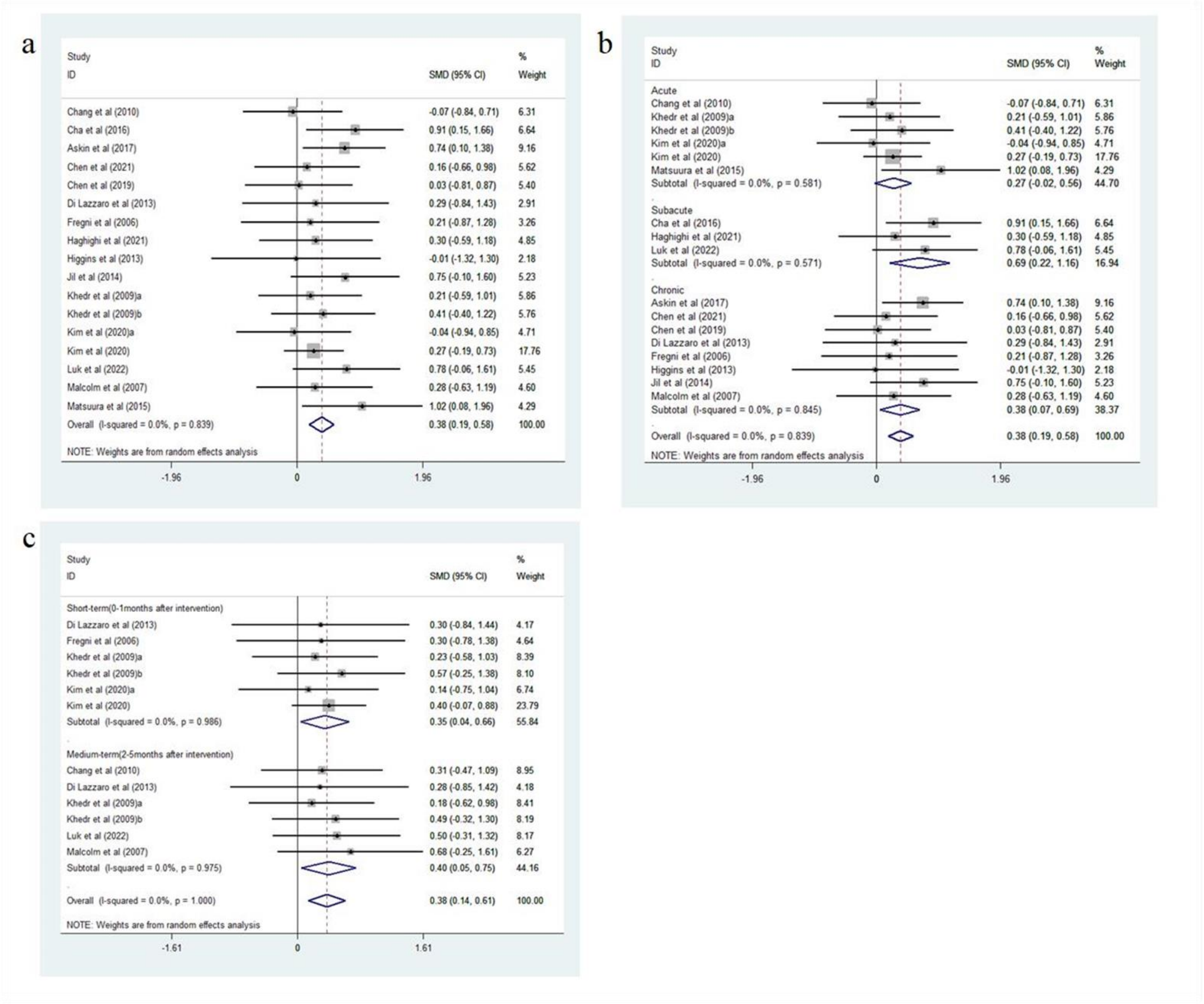

Supplementary Fig.1 a. Forest plot of hand function recovery in stroke patients compared with controls; b. Forest plot of hand function recovery in stroke patients disaggregated by phase of stroke compared with controls; c. Forest plot of hand function recovery in stroke patients disaggregated by measurement time compared with controls.

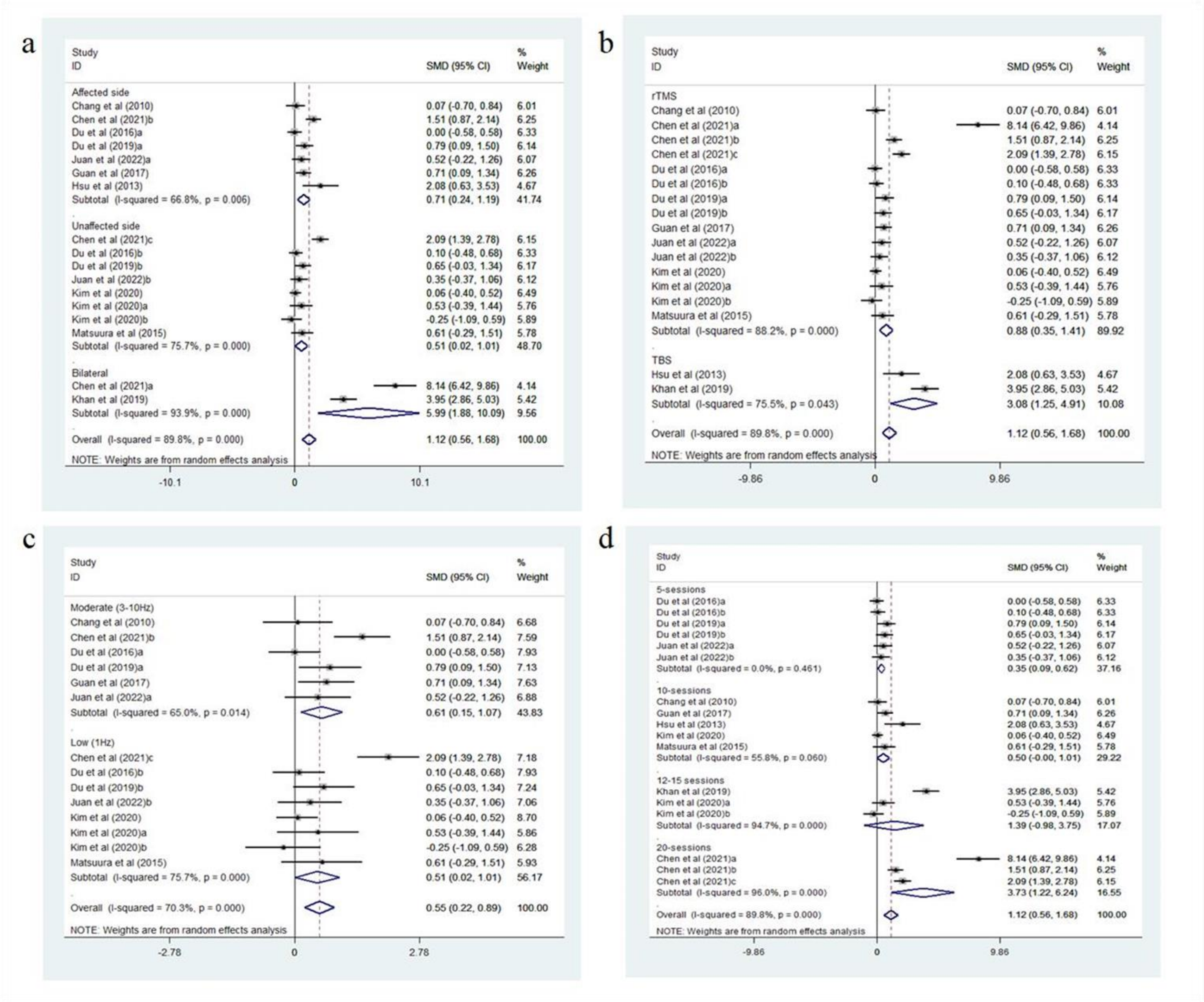

Supplementary Fig.2 a. Forest plot of FMA-UE compared with controls by stimulation site in patients with acute phase stroke; b. Forest plot of FMA-UE in patients with acute phase stroke disaggregated by stimulation method compared with controls; c. Forest plot of FMA-UE in patients with acute phase stroke disaggregated by by rTMS frequency compared with controls; d. Forest plot of FMA-UE in patients with acute phase stroke disaggregated by by number of treatment sessions compared with controls.

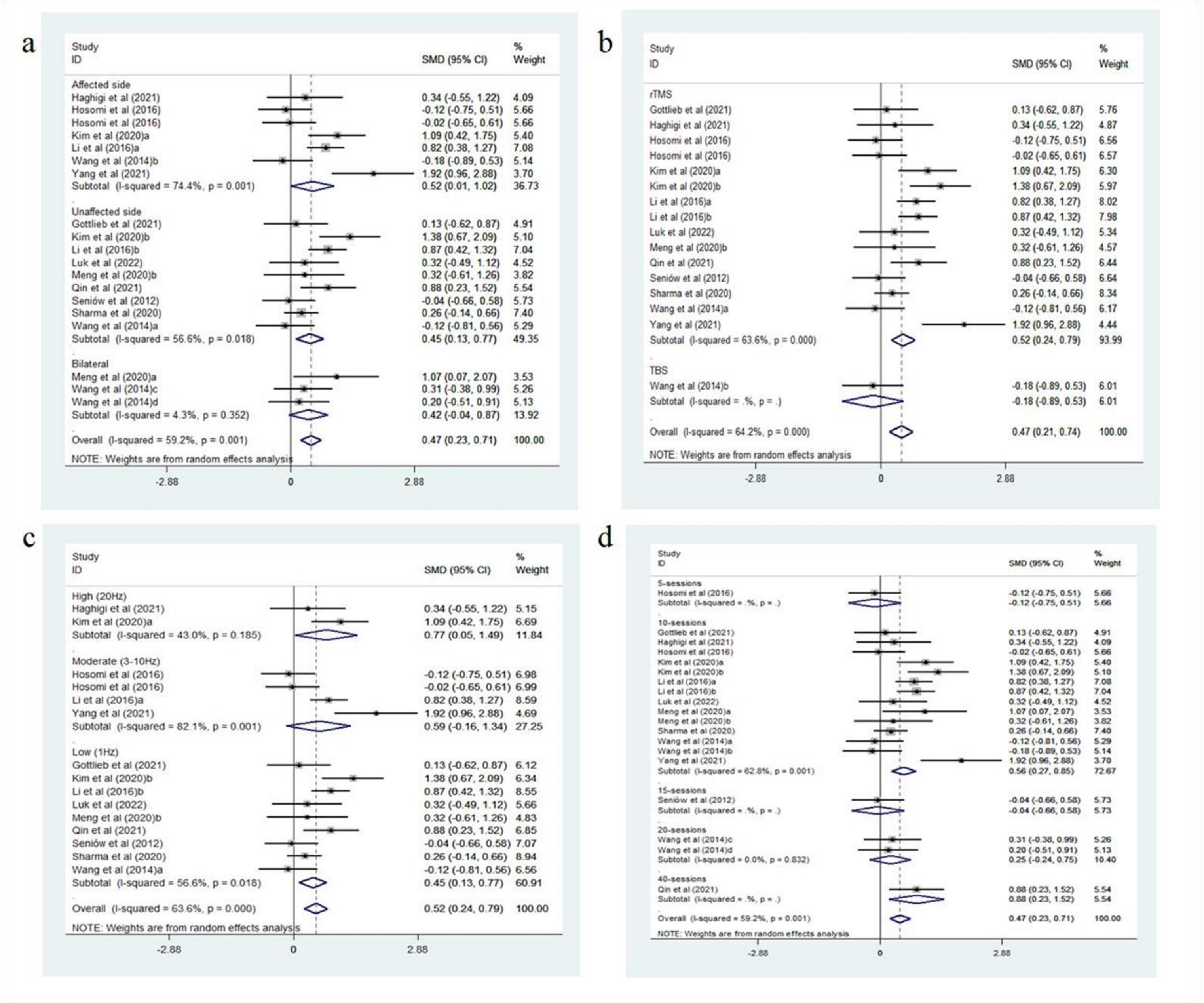

Supplementary Fig.3 a. Forest plot of FMA-UE in patients with subacute phase stroke disaggregated by stimulation site compared with controls; b. Forest plot of FMA-UE in patients with subacute phase stroke disaggregated by stimulation method compared with controls; c. Forest plot of FMA-UE in patients with subacute phase stroke disaggregated by by rTMS frequency compared with controls; d. Forest plot of FMA-UE in patients with subacute phase stroke disaggregated by by number of treatment sessions compared with controls.

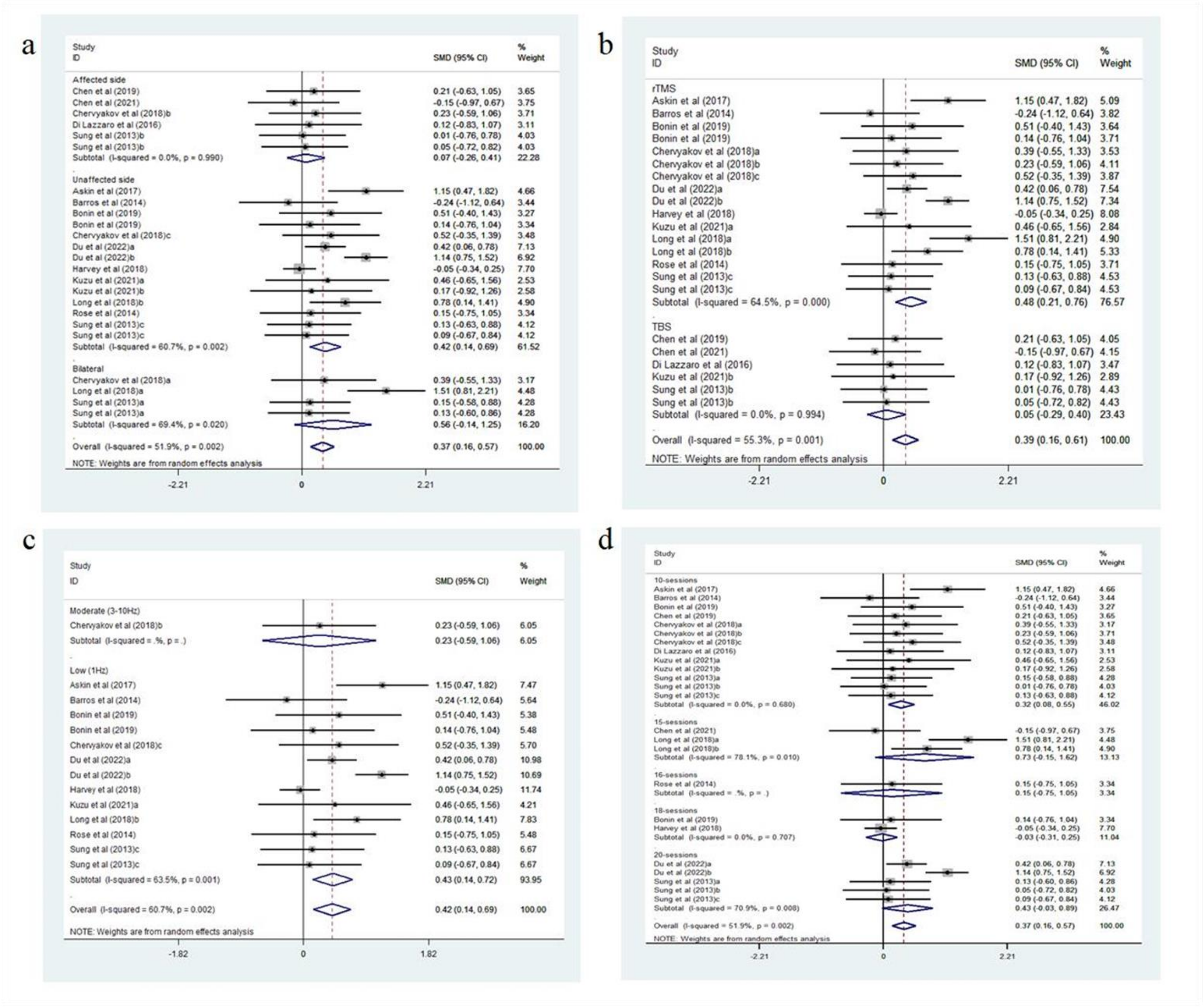

Supplementary Fig.4 a. Forest plot of FMA-UE in patients with chronic phase stroke disaggregated by stimulation site compared with controls; b. Forest plot of FMA-UE in patients with chronic phase stroke disaggregated by stimulation method compared with controls; c. Forest plot of FMA-UE in patients with chronic phase stroke disaggregated by by rTMS frequency compared with controls; d. Forest plot of FMA-UE in patients with chronic phase stroke disaggregated by by number of treatment sessions compared with controls.

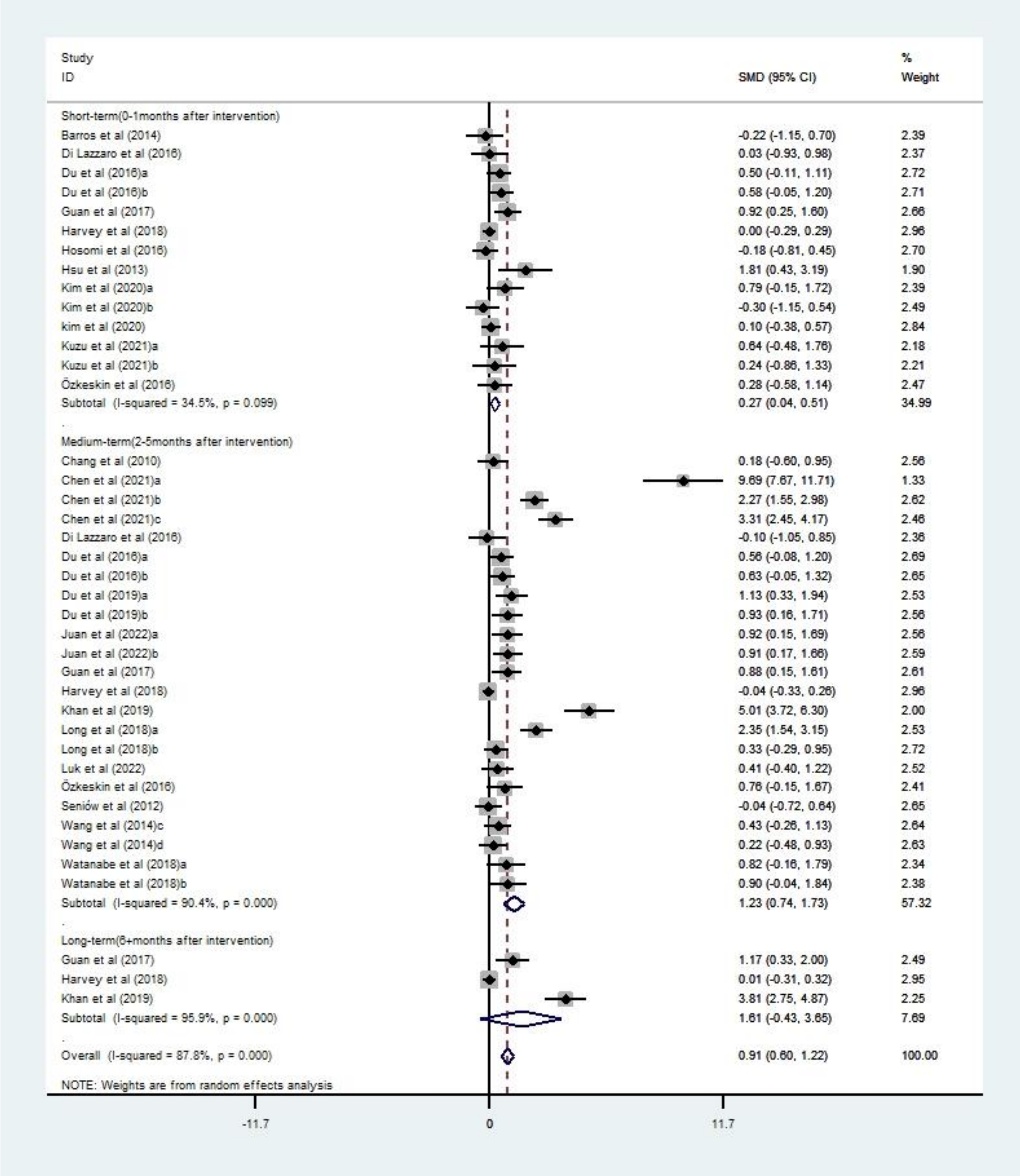

Supplementary Fig.5 Forest plot of FMA-UE in stroke patients disaggregated by time of assessment compared with controls.

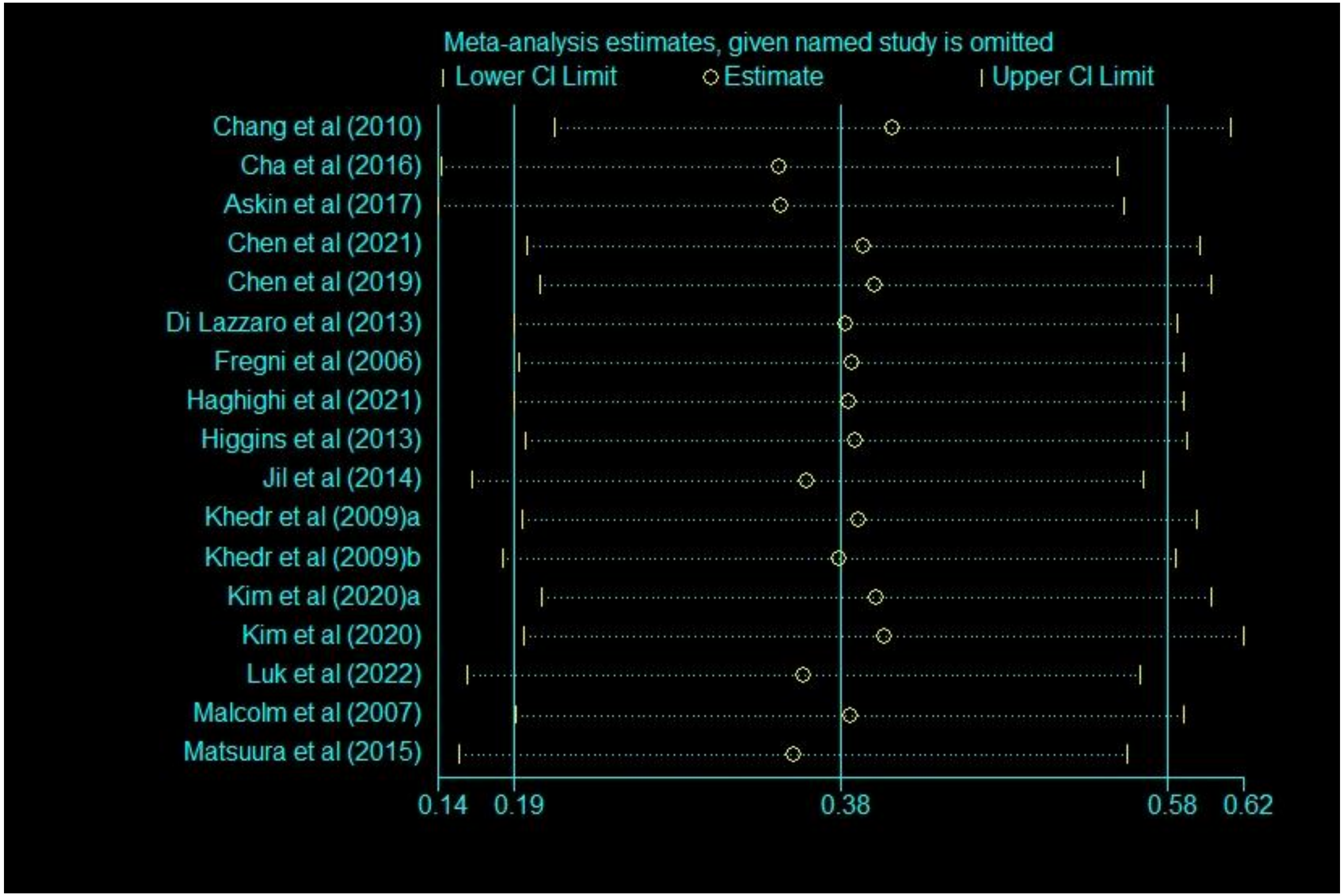

Supplementary Fig.6 Results of sensitivity analysis showing the stability of the results in the included studies
